# REPLY TO THE AUTHORS: Re: The effect of urethroplasty surgery on erectile and orgasmic functions: a prospective study

**DOI:** 10.1590/S1677-5538.IBJU.2018.0793.1

**Published:** 2019-04-01

**Authors:** Ahmet Urkmez, Ozgur H. Yuksel, Emrah Ozsoy, Ramazan Topaktas, Aytac Sahin, Orhan Koca, Metin I. Ozturk

**Affiliations:** 1 Department of Urology, University of Health Sciences, Haydarpasa Numune Research & Training Hospital, Istanbul, Turkey; 2 Department of Urology, University of Health Sciences, Fatih Sultan Mehmet Research & Training Hospital, Istanbul, Turkey

*To the editor,*

We would like to thank the author of the letter to the editor for her or his correct point that the lack of validated questionnaires specific in the field of urethral stricture and urethroplasty (1). Today, many questionnaires available to put towards the diagnosis of erectile dysfunction and evaluate the success of treatment. International Index of Erectile Function (IIEF), was first used in the clinical study of sildenafil and is now the most commonly used assessment questionnaire in the evaluation of erectile dysfunction with subsequent validations (2). However, the IIEF evaluation provides minimal information about the ejaculatory and orgasmic function of patients (3). Besides, validation studies on questionnaires for assessing erectile and ejaculatory functions are ongoing, even by country and language (4-6).

Sangkum et al. have reported that most patients with urethral stricture complained of sexual dysfunction, especially about reduced ejaculatory fluid (85%) and after urethroplasty, no one reported a worsening erection; and many of them reported that there was a significant improvement in erection, ejaculation, relationship with their partner, sexual activity and desire (7). Also, Erickson et al., have found that urethral reconstructive surgery affects ejaculatory functions positively while not significantly affecting erectile function or sexual dysfunction (8). Since we did not have a validated questionnaire in patients with urethral stricture, we used the IIEF questionnaire, which is the most widely used validated test in the world. Recently, case- based or diseased- based questionnaires have been increasingly used. Accordingly, there are studies related to special questionnaires for patients with urethral stricture (9, 10). But these are not validated. As stated by the author of the letter to the editor, studies in this field should continue to better investigate the effect of urethral stricture and urethroplasty surgery on orgasmic and ejaculatory functions.
